# Quality assessment of paediatric randomized controlled trials published in China from 1999 to 2022: a cross-sectional study

**DOI:** 10.1186/s12887-024-04839-3

**Published:** 2024-05-27

**Authors:** Bennian Huo, Song Xu, Yao Liu, Lin Su, Yuntao Jia, Maolin Ai, Nange Yin, Lin Song

**Affiliations:** 1 Department of Pharmacy Children’ s Hospital of Chongqing Medical University, National Clinical Research centre for Child Health and Disorders, Ministry of Education Key Laboratory of Child Development and Disorders, Chongqing Key Laboratory of Pediatrics, Chongqing Clinical pharmacy Key Specialty Construction Project, Chongqing, China; 2 Department of Orthopedics, Bishan Traditional Chinese Medicine Hospital, Chongqing, 400014 China; 3 Department of Pharmacy, Daping Hospital, Army Medical University, Chongqing, China

**Keywords:** Paediatric, Randomized controlled trial, Characteristics, Quality assessment, China

## Abstract

**Background:**

Randomized controlled trials (RCTs) are usually the basis of evidence-based medicine, but whether the results of RCTs can be correctly translated into clinical practice depends on the quality of the literature reported. In this study, we evaluated the general characteristics and quality of paediatric RCTs published in China to provide evidence for the reporting of paediatric RCTs and their application in clinical practice.

**Methods:**

We conducted a cross-sectional observational study of paediatric RCTs published in paediatric journals in China between January 1, 1999, and December 30, 2022. All RCTs that included children (younger than 18 years old) were retrieved, and the general characteristics of the RCTs were extracted and analysed. The quality of the RCTs was assessed by the Cochrane quality assessment protocol.

**Results:**

After screening 20 available paediatric journals, 3545 RCTs were included for analysis. The average annual growth rate of the number of published paediatric RCTs from 1999 to 2022 was 7.8% (*P* = 0.005, R^2^ = 0.311). Most of the studies were carried out in East China [1148 (32.4%]; the centres of the RCTs were mainly single-centre [3453 (97.4%], and the interventions were mainly medication [2442 (68.9%)]. Comparing RCTs published in 2017–2022 with RCTs published in 1999–2004, the quality of RCTs significantly improved in terms of random sequence generation, allocation concealment, blinding participants and personnel, incomplete outcome data and selective outcome reporting. RCTs published in multiple centres from the Chinese Science Citation Database were identified, and the approval of the ethics committee was of better quality for all the analysed risk of bias items.

**Conclusion:**

The number and quality of paediatric RCTs reported in China have improved in recent years, but the overall quality was relatively low. Special attention should be given to allocation concealment and blinding outcome assessment, and dropouts, adverse effects and sample size calculations should be reported. Promoting government policies, strengthening the standardization of journal publishing and advancing the registration of clinical trials are feasible measures.

**Supplementary Information:**

The online version contains supplementary material available at 10.1186/s12887-024-04839-3.

## Background

Due to a lack of sufficient information on children’s medication on drug labels and difficulties in conducting clinical trials [1, 2], off-label prescribing in children is common globally and in China [3]. Overall, the off-label prescribing rates in children ranged from 46.9 to 98.1% in China [1, 4–10], 53.0–98.1% in neonates [1, 5, 6], 57.2–82.7% in outpatients [7, 8] and 48.9–79.0% [9, 10] in inpatients; thus, increased risks related to treatment and legal practices exist in the treatment of children’s diseases [4]. In addition to drug labels, clinical treatment guidelines and expert consensuses based on clinical research have become the main sources and basis of physicians’ medication evidence [11], especially paediatricians, and the clinical research evidence in the guidelines and expert consensuses are generally graded according to the research design type. Therefore, with the introduction of evidence-based medicine, paediatricians and clinicians are requested to make clinical decisions through scientific evidence. However, the clinical applicability of the research results is related not only to the design of the research but also to many other factors, especially the factors that may reflect the reliability of the research results.

Randomized controlled trials (RCTs) are considered the best research protocol for assessing the effectiveness and safety of interventions and have been defined in many guidelines and expert consensuses as evidence of high quality and given a corresponding clinical application recommendation grade [12, 13]. However, whether the results of RCTs can be correctly translated into clinical practice also depends on the quality of the literature reported. Previous studies have shown that there are limited published clinical trials about children, especially randomized controlled trials and multiple-centre studies [14], and most of the paediatric RCTs were published with high or unclear risk of bias in different ways [15]. As is evident from the literature [16], low-quality RCTs can hinder the reader’s objective assessment of bias and lead to false estimates of the effect of the intervention, which may lead to harmful clinical decisions.

In addition, in recent years, clinical trials in the paediatric population have garnered increased attention in China. In 2011, the government proposed encouraging the research and development and production of drugs for children, and since then, a number of documents or measures have been issued to encourage clinical trials in paediatrics, such as the National Program for Child Development in China (2011–2020) in 2011 [17], the basic principles for the evaluation of varieties of drugs for children in urgent need in 2015 [18], the Technical Guidelines for Drug Clinical Trials in Pediatric Population in 2016 [19] and the Technical Guidelines for Pharmacokinetic Research in Pediatric Population in 2020 [20]. With these policies, the number of industry-sponsored paediatric clinical studies in China has increased, and we found that the number of paediatric clinical studies reported in the literature, including investigator-initiated clinical trials, is also increasing significantly. One study analysed the quality of paediatric RCTs in China before 2011 [21], but we found that the included RCTs were not comprehensive enough, and general characteristics such as the characteristics of the investigators, geographical distribution of the trials, and ethical characteristics were not reported.

All clinical trials must be registered at a clinical trial registry to be eligible for publication [22]. In recent years, several databases containing registries of clinical trials have been established to organize information and to facilitate access to the general public and government authorities, such as the ClinicalTrials.gov database, the International Clinical Trials Registry Platform and the Chinese Clinical Trial Registry databases. Clinical trial registration could improve the transparency of clinical trial methods and results and was also a marker for reducing the risk of bias [23], but trial discontinuation and nonpublication were common among interventional trials conducted in children [24], and more than one-third of RCTs completed in newborns might not have yet been published [25]. In 2021, we analysed the general characteristics and identified the reasons for and factors associated with clinical trial discontinuation in mainland China. In that study, the common reasons for trial discontinuation were commercial or strategic decisions [84 (26.9%)] and futility/lack of efficacy [70 (22.4%)] [26]. In 2022, we analysed paediatric clinical trials conducted in China and reported that the current challenge was the further development of dosage forms suitable for children with special attention to neonates and premature infants and the improvement of the uneven geographical distribution of sponsors and researchers [27].

Thus, based on previous research, the objective of this study was to determine the general characteristics and quality of paediatric randomized controlled trials published in mainland China over the decade 1999–2022 by assessing trials published in all paediatric journals in China to evaluate the quality trends in paediatric clinical trials over the decades and to provide a reference for the development and reporting of paediatric clinical research and its application in clinical practice as evidence.

## Methods

### Selection of journals and RCTs

We searched the currently available paediatric journals in mainland China from the following databases on 26 February 2023: China National Knowledge Infrastructure (https://www.cnki.net), China Science and Technology Journal Database (https://qikan.cqvip.com), Wanfang Data Knowledge Service Platform (https://www.wanfangdata.com.cn) and China Biology Medicine disc (www.sinomed.ac.cn). Journals that were classified as paediatric journals were included, and we excluded English journals and popular science periodicals. A total of 20 paediatric medical journals were included in this study, namely, the *Chinese Journal of Applied Clinical Pediatrics, the Chinese Journal of Pediatrics, the Chinese Journal of Contemporary Pediatrics, the Journal of Clinical Pediatrics, the Chinese Journal of Pediatric Surgery, the Chinese Journal of Practical Pediatrics, the Chinese Journal of Evidence-Based Pediatrics, the Chinese Journal of Neonatology, the Journal of Pediatrics of Traditional Chinese Medicine, Women’s Health Research, the Journal of Clinical Pediatric Surgery, the Chinese Pediatric Emergency Medicine, the Chinese Journal of Obstetrics & Gynecology and Pediatrics (Electronic Edition), the Chinese Pediatrics of Integrated Traditional and Western Medicine, the Journal of China Pediatric Blood and Cancer, the Chinese Journal of Child Health Care, the International Journal of Pediatrics, the Journal of Pediatric Pharmacy, Maternal and Child Health Care of China, and the Journal of Developmental Medicine (Electronic Version).* The first 7 journals are included in the Chinese Science Citation Database (CSCD), which is the Chinese equivalent of the Science Citation Index (SCI), represents the most influential journal in the field of natural sciences [28], and has earned a good reputation among Chinese scientists [29]. The remaining 13 journals were non-CSCD journals.

Two authors independently screened the titles, abstracts or full texts of all the studies published in the 20 journals from 1999 to 2022, and any disagreements were resolved through discussion or by consulting a third author. Trials were considered for inclusion if all the participants were less than 18 years of age and if the randomization method was used to assign participants to different intervention groups, regardless of whether the exact randomization method was used, and only Chinese language studies were included. We excluded overviews, meta-analyses, clinical treatment guidelines, expert consensuses, and conference proceedings.

### Data extraction and quality assessment

Two authors reviewed the full texts of all the included trials, extracted the study characteristics and performed quality assessments, and any disagreements were resolved through discussion or by consulting a third author. We used a data collection form that had been piloted on fifty studies and included the following items:

1. General characteristics: journal name, publication date, first affiliation of the authors and province of the principal investigator located. The provinces were divided into seven regions according to China’s seven geographical divisions, including north, east, south, central, northeast, northwest and southwest.
2. Ethical characteristics: Ethical approval and informed consent were obtained.
3. Trial characteristics: Number of research centres (multiple-centre or single-centre trial), funding resources, trial registration information, intervention, control, studying diseases, sample size, and length of follow-up. In this study, the disease categories were coded according to the International Statistical Classification of Diseases and Related Health Problems, Tenth Revision, International Classification of Diseases (ICD)-10 classification [30], and the categories of major diseases were based on the “Guidelines for the Use of Disease Definitions for Major Diseases Insurance” (2020 revision) issued by the China Insurance Association and Chinese Medical Doctor Association in November 2020 [31]. The categories of rare diseases were based on the “first Batch of Rare Diseases Catalogue”, which was issued by the National Health Commission of China in May 2018 [32]. In addition, we recorded whether the study reported a method for calculating the sample size, whether the study reported the comparability of baseline characteristics, whether dropouts and adverse events were reported, and whether conflicts of interest were stated.
4. Quality assessment: The quality evaluation method was based on the Cochrane Collaboration’s tool for assessing risk of bias [33], including the following 7 items: random sequence generation, allocation concealment, blinding of participants and personnel, blinding of outcome assessment, incomplete outcome data, selective outcome reporting, and other bias. We judged each potential source of bias as high, low, or unclear. The data collection form and details of the quality assessment of the risk of bias are shown in Supplementary Tables 1 and Supplementary Table 2, respectively.

### Statistical analysis

Descriptive analyses were used to summarize the data, and frequency (percentage) was used for qualitative data. A simple regression model was used to analyse the trends in the number of RCTs included, with *P* < 0.05 indicating a statistically significant difference. To evaluate whether the quality of the RCTs improved over time, we divided all the RCTs into four time strata based on the year of publication: “1999–2004”, “2005–2010”, “2011–2016”, and “2017–2022”. Binary logistic regression analysis was performed to explore the relationship between each criterion and time. The publication year was used as a continuous variable, and the publication year was used as a categorical variable. The years 1999–2004 were used as reference strata. We used “low risk of bias” or “yes” as the reference category, and compared to “high risk of bias” or “not reported”, we reported odds ratios (ORs) with 95% confidence intervals (CIs). The chi-square test and Fisher’s exact test were used for proportions, and the risk of bias domains and differences among subgroups were compared. A *P* value less than 0.05 was considered to indicate statistical significance. All the statistical analyses were performed on a personal computer with the statistical package SPSS for Windows (version 25.0).

## Results

### Publication time trends and geographical distribution of the RCTs

**Fig. 1:**
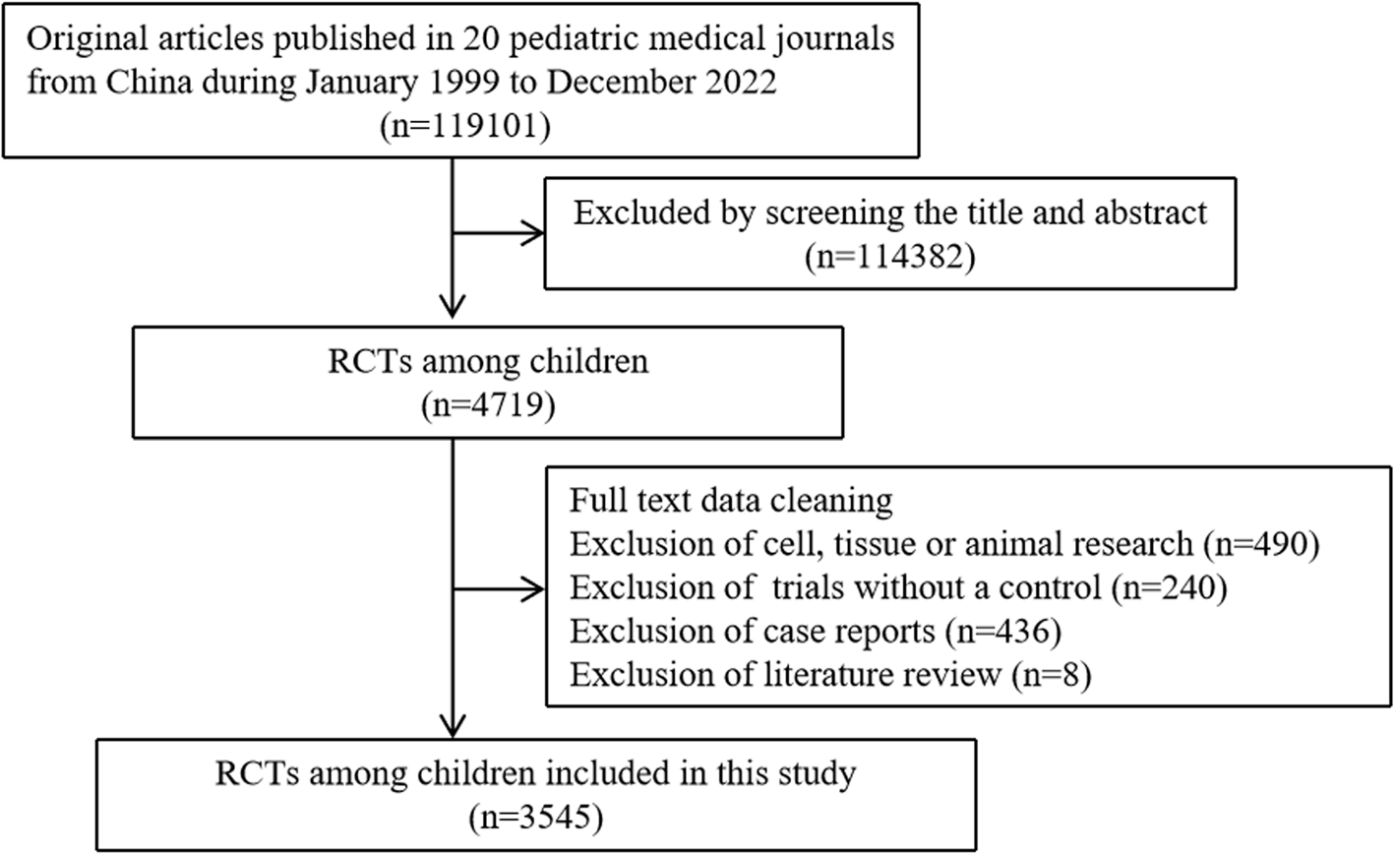
Flow chart of trial selection

From January 1999 to December 2022, 119,101 articles were published in 20 Chinese paediatric journals. After screening the study design and participants, 3545 of the studies were selected for inclusion and data analysis (Fig. 1). The majority of the included studies were published on *Maternal and Child Health Care* in China [713 (20.1%)], followed by the *Journal of Pediatrics of Traditional Chinese Medicine* [634 (17.9%] and the *Journal of Pediatric Pharmacy* [528 (14.9%)], which are Non-CSCD journals.

**Fig. 2:**
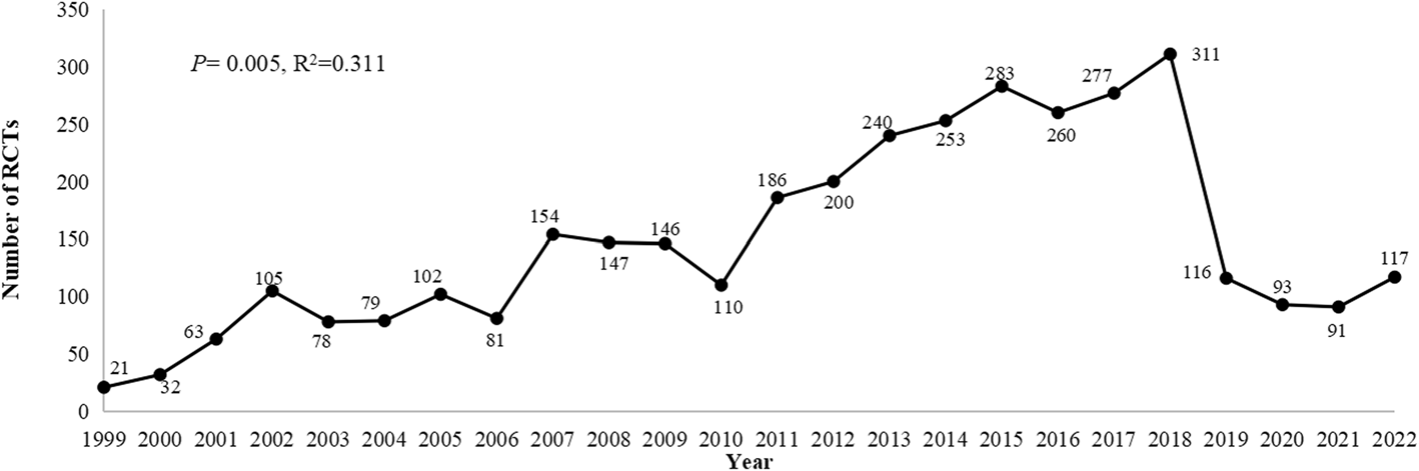
Annual number of paediatric RCTs in China from 1999 to 2022

**Fig. 3:**
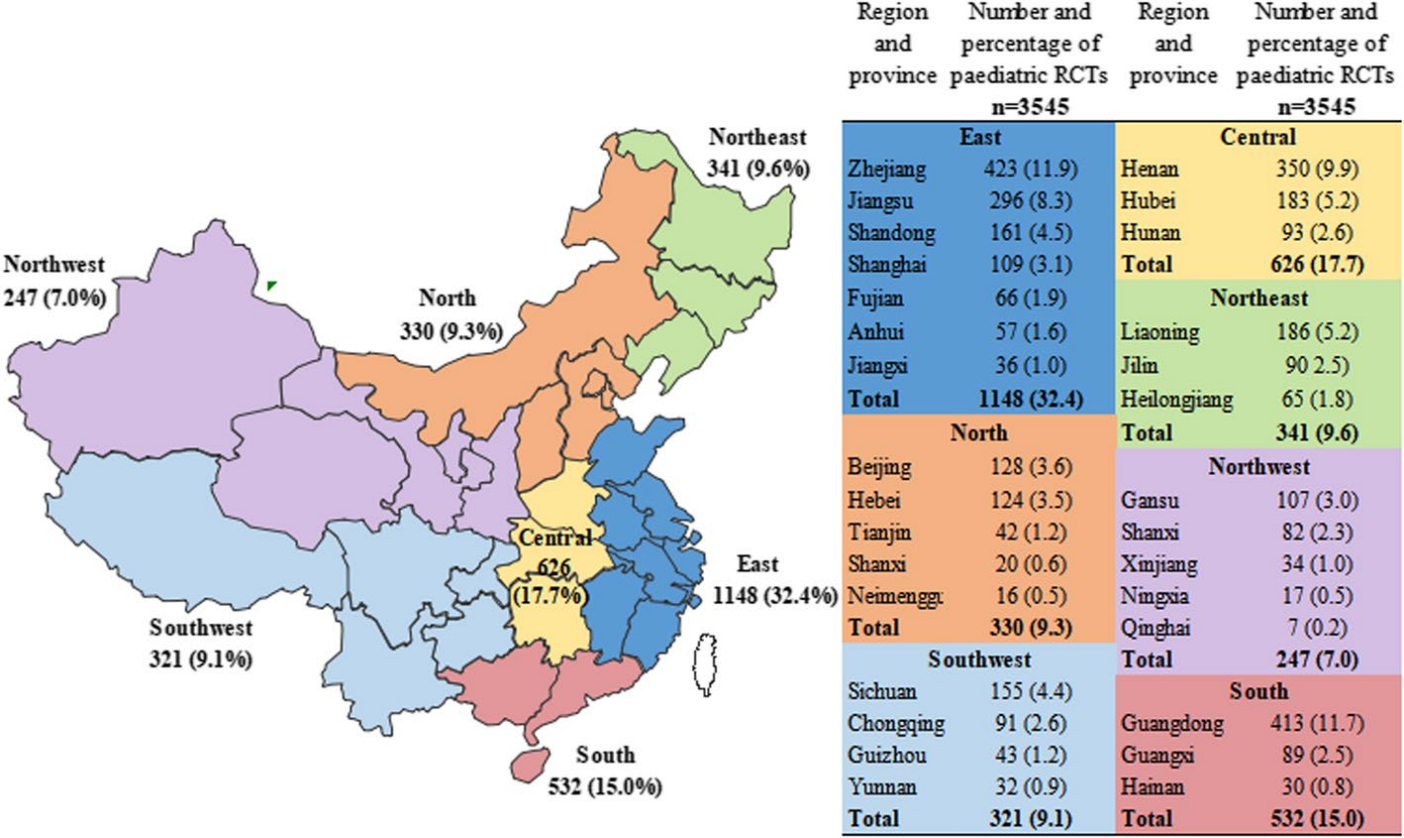
Geographical distribution of paediatric RCTs in China from 1999 to 2022 (the map depicted in Figure 3 was created by our engineers from the information centre using Excel 2010)

A simple regression model revealed that the average annual growth rate of the number of trials from 1999 to 2022 was 7.8% (*P* = 0.005, R^2^ = 0.311). The number of RCTs increased in 2011, and a notable increase occurred in 2018, with 311 RCTs published, corresponding to an increase of 67.2% over the number published in 2011. Since 2019, the number of published RCTs began to gradually decrease (Fig. 2). Geographical distribution analysis revealed that all the published RCTs were carried out in 30 different cities in China (Fig. 3). For multiple-centre RCTs, the cities where the coordinating investigators were located were included in the analysis. Most of the studies were carried out in East China [1148 (32.4%)], followed by Central [626 (17.7%)] and South [532 (15.0%)], and over one-third of the RCTs were conducted in Zhejiang, Henan and Guangdong. The most prolific institutions were Beijing Children’s Hospital Capital Medical University [95 (2.7%)], followed by Hunan Children’s Hospital [81 (2.3%)] and Henan Children’s Hospital [72 (2.0%)].

### Characteristics of the RCTs

The characteristics of the included trials are shown in Table 1. Most of the RCTs [2781 (78.4%)] were conducted at teaching hospitals; only 2.6% (92/3545) of the RCTs were conducted at multiple centres, with the number of centres ranging from 2 to 11 (average = 6.5); and 15.2% (539/3545) of the RCTs reported funding resources, of which only 10 were subsidized by companies. A total of 18.6% (659/3545) of the authors stated that the RCT was approved by the ethics committee, and 40.0% (1419/3545) of the authors stated that the patients signed the informed consent. Very few trials have shown that registration has been carried out on relevant websites. Most of the related research has been conducted on medication, and only a few studies have used a placebo as a control. The median sample size of the RCTs was 86 (range from 11 to 480), but only 2% (71/3545) of them reported the sample size calculation process. Most of the RCTs [3294 (92.9%)] reported the comparability of the baseline characteristics of the different groups but did not report the total follow-up time, number of dropouts, or number of conflicts of interest, and less than half of the RCTs [1523 (43.0%)] reported adverse events.

**Table 1:** General characteristics of 3545 paediatric randomized controlled trials in China

Items	Frequency (%)
**Journal**	
CSCD	730 (20.6)
Non-CSCD	2815 (79.4)
**Authors’ affiliation**	
Teching hospotital	2781 (78.4)
Non-teching hospital	654 (18.4 )
Scientific research institutions	110 (3.1)
**Trial centre**	
Multiple-centre	92 (2.6)
Single-centre	3453 (97.4)
**Funding resources**	
Company	10 (0.3)
National	62 (1.7)
Provincial	467 (13.2)
Not-reported	3006(84.8)
**Ethical approval**	
Approved	661 (18.6)
Not-reported	2884 (81.4)
**Informed consent**	
Signed informed consent	1419 (40.0)
Not-reported	2126 (60.0)
**Trial registration information**	
Registered	29 (0.8)
Not-reported	3516 (99.2)
**Types of intervention**	
Medication	2442 (68.9)
Surgical	377 (10.6)
Rehabitation	385 (10.9)
Others	341 (9.6)
**Types of control**	
Active medicine	2386 (67.3)
Blank control	907 (25.6)
Dose-response control	197 (5.6)
Placebo control	55 (1.6)
**Sample size**	
≤ 100	2268 (64.0)
100–500	1236 (34.9)
≥ 500	41 (1.2)
**Follow-up time**	
≤1 year	360 (10.2)
1-3year	178 (5.0)
≥ 3year	21 (0.6)
Not-reported	2986 (84.2)
**Comparability of baseline**	
Comparable	3294 (92.9)
Not-reported	251 (7.1)
** Report of sample size calculation done prior to study initiation**	71 (2.0)
** Report of adverse events**	1523 (43.0)
** Report of dropouts**	328 (9.3)
** Report of conflict of interest**	582 (16.4)

*CSCD* Chinese Science Citation Database

The distribution of the studied diseases among the included studies, categorized according to the (ICD)-10 classification, is shown in Table 2. Diseases of the respiratory system account for more than one-third [1278 (36.1%)] of all identified diseases, followed by certain conditions originating in the perinatal period [414 (11.7%)], diseases of the digestive system [358 (10.1%)] and certain infectious and parasitic diseases [345 (9.7%)]. Paediatric asthma and *Mycoplasma pneumoniae* were the most commonly studied diseases, with 10.6% (374/3545) and 9.1% (322/3545) of the trials, respectively. In terms of disease types, only 2.3% (80/3545) of the RCTs investigated major diseases, including congenital heart disease (*n* = 34), acute lymphoblastic leukaemia (*n* = 10), paediatric tumours (*n* = 9), systemic lupus erythematosus (*n* = 9), etc., and no identified RCTs involved rare diseases.

**Table 2:** Distribution of disease categories of paediatric randomized controlled trials published in China from 1999 to 2022 (*n* = 3545)

Disease categories^a^	Frequency (%)
Respiratory system	1278 (36.1)
Certain conditions originating in the perinatal period	414 (11.7)
Digestive system	358 (10.1)
Infectious and parasitic diseases	345 (9.7)
Nervous system	179 (5.0)
Blood, blood-forming organs, immune mechanism	160 (4.5)
Mental and behavioral disorders	147 (4.1)
Symptoms, signs and abnormal clinical and laboratory findings, not elsewhere classified	119 (3.4)
Endocrine, nutritional and metabolic diseases	93 (2.6)
Genitourinary system	88 (2.5)
Factors influencing health status and contact with health services	83 (2.3)
Musculoskeletal system and connective tissue	78 (2.2)
Circulatory system	74 (2.1)
Skin and subcutaneous tissue diseases	55 (1.6)
Others^b^	74 (2.1)

^a^Disease categories were coded by International Classification of Diseases (ICD)-10 classification

^b^Other disease categories including congenital malformations, deformations and chromosomal abnormalities, eye and appendix diseases, injury, poisoning and certain other consequences of external causes, neoplasms, and ear and mastoid process

### Quality assessment of the RCTs

The results of the quality assessment based on the Cochrane Collaboration methods for risk assessment are shown in Table 3. A total of 35.9% (1272/3545) of the RCTs reported a truly random method, 3.1% (109/3545) of the RCTs used adequate methods for allocation concealment, 4.2% (150/3545) of the RCTs ensured the blinding of participants and personnel, and 1.5% (52/3545) of the RCTs ensured the blinding of outcome assessment. 45.8% (1624/3545) RCTs showed “low risk of bias” of incomplete outcome data, of which 9.3% (151/1624) RCTs reported dropouts, but only 5.2% (84/1624) RCTs used intention-to-treat analysis. A total of 24.6% (873/3545) of the RCTs were judged as having obvious selective outcome reporting because the outcome that was explicitly reported in the methodology was not shown in the results. For other bias, very few studies [85 (2.4%)] were considered to have a low risk of bias, and they were mostly registered and company-funded trials.

**Table 3:** Quality assessment of 3545 paediatric randomized controlled trials in China

Items	Risk of bias assessment	Frequency (%)
**Method of random sequence generation**	**Low risk**	1272 (35.9)
	Referring to a random number table	1046 (29.5)
	Using a computer random number generator;	115 (3.2)
	Drawing of lots	95 (2.7)
	Coin tossing	14 (0.4)
	Throwing dice	2 (0.1)
	**High risk** Randomized according to the odd or even date of birth; date of admission; or hospital or clinic record number, etc.	511 (14.4)
	**Unclear** Randomization was stated, but the process was not described	1762 (49.7)
**Method of allocation concealment**	**Low risk**	109 (3.1)
	Central allocation	55 (1.6)
	Sealed opaque envelops	32 (0.9)
	Sequentially numbered identical drug containers	22 (0.6)
	**High risk** Participants or the investigators enrolling participants could potentially predict the assignments	403 (11.4)
	**Unclear** Method of concealment was not described or not described in sufficient detail	3033 (85.6)
**Blinding of participants and personnel**	**Low risk** Blinding of participants and key study personnel was ensured	150 (4.2)
	**High risk** Open label; no blinding or incomplete blinding; or attempted blinding of key study participants and personnel, but it was likely that the blinding was compromised	236 (6.7)
	**Unclear** Insufficient information for a clear decision	3159 (89.1)
**Blinding of outcome assessment**	**Low risk** Blinding of outcome assessment was ensured	52 (1.5)
	**High risk** Open label, no blinding of outcome assessment and the outcome measurement was likely to be influenced by lack of blinding	99 (2.8)
	**Unclear** Insufficient information for a clear decision	3394 (95.7)
**Incomplete outcome data**	**Low risk** No missing data or missing data be analyzed properly	1624 (45.8)
	**High risk** Missing data not be analyzed properly	1194 (33.7)
	**Unclear** Insufficient information for a clear decision	727 (20.5)
**Selective outcome reporting**	**Low risk** All outcomes reported are included in the analysis	363 (10.2)
	**High risk** Not report pre-specified primary outcomes or key results from such studies	873 (24.6)
	**Unclear** Insufficient information for a clear decision	2309 (65.1)
**Other bias**	**Low risk** For registered trials, the study appears to be free of other sources of bias	85 (2.4)
	**High risk** The subject’s baseline data not present or incomparable, and had a potential source of bias related to the specific study design use	258 (7.3)
	**Unclear** Insufficient information for a clear decision	3202 (90.3)

The quality evaluation method was based on the Cochrane Collaboration methods for risk assessment [33]

### Analysis of trends in quality and influencing factors

Table 4 shows the trends of the quality of the RCTs over time when using “low risk of bias” or “yes” as the reference category and time stratum “1999–2004” as the reference time stratum. We identified that there was a statistically significant improvement in the odds for low risk of bias for five items (random sequence generation, allocation concealment, blinding of participants and personnel, incomplete outcome data and selective outcome reporting). For other related quality assessment items, two items (ethical approval and signed informed consent) significantly improved over time.

**Table 4:** Results from the logistic regression analysis for low risk of bias or other related quality assessments of paediatric randomized controlled trials in China over time

Items^a^	P0ublication year^b^		Time-periods of publication year^c^						
			1999–2004	2005–2010		2011–2016		2017–2022	
	OR (95% CI)	*P*-value	Ref^d^	OR (95% CI)	*P*-value	OR (95% CI)	*P*-value	OR (95% CI)	*P*-value
**Risk of bias assessment**									
Random sequence generation	3.500 (2.987-4.100)	< 0.000	Ref	3.547 (2.017–6.236)	< 0.000	8.919 (5.339–14.900)	< 0.000	50.540 (28.069–91.002)	< 0.000
Allocation concealment	4.363 (3.062–6.217)	< 0.000	Ref	3.911 (1.095–13.974)	0.036	1.807 (0.513–6.359)	0.357	40.150 (11.859-135.937)	< 0.000
Blinding participants and personnel	1.611 (1.311–1.980)	< 0.000	Ref	4.046 (1.828–8.955)	0.001	4.586 (2.045–10.283)	< 0.000	6.850 (3.068–15.294)	< 0.000
Blinding outcome assessment	1.449 (1.026–2.047)	0.035	Ref	1.939 (0.488–7.702	0.347	1.986 (0.469–8.417)	0.352	3.792 (0.949–15.151)	0.059
Incomplete outcome data	2.004 (1.838–2.184)	< 0.000	Ref	1.272 (0.941–1.720)	0.117	2.928 (2.223–3.858)	< 0.000	6.486 (4.843–8.686)	< 0.000
Selective outcome reporting	1.677 (1.479–1.902)	< 0.000	Ref	0.947 (0.616–1.454)	0.802	1.149 (0.768–1.717)	0.500	3.966 (2.699–5.830)	< 0.000
Other bias	2.440 (1.883–3.162)	< 0.000	Ref	1.144 (0.469–2.792)	0.767	2.050 (0.838–5.015)	0.116	10.789 (4.944–23.545)	< 0.000
**Other related quality assessments**									
Ethical approval	3.573 (3.122–4.090)	< 0.000	Ref	0.822 (0.444–1.523)	0.534	3.256 (1.956–5.418)	< 0.000	14.122 (8.542–23.345)	< 0.000
Signed informed consent	3.195 (2.896–3.524)	< 0.000	Ref	1.272 (0.785–2.061)	0.328	12.597 (8.284–19.156)	< 0.000	22.510 (14.708–34.451)	< 0.000
Report of withdraws and dropouts	1.075 (0.951–1.215)	0.250	Ref	1.219 (0.804–1.850)	0.351	0.536 (0.353–0.812)	0.003	1.396 (0.942–2.070)	0.097
Adverse effects described	1.004 (0.936–1.078)	0.905	Ref	0.800 (0.621–1.031)	0.085	0.755 (0.599–0.951)	0.017	0.934 (0.734–1.187)	0.934
Sample size calculation done prior to study initiation	0.824 (0.647–1.048)	0.115	Ref	0.638 (0.314–1.298)	0.215	0.244 (0.115–0.517)	< 0.000	0.628 (0.323–1.221)	0.170

*OR* Odds ratio, *CI* Confidence interval

^a^Low risk vs. high risk of bias; or yes vs. not-reported

^b^Time was entered in the logistic regression model as a continuous variable

^c^Time was entered in the logistic regression model as a categorical variable

^d^The Ref means taking publication year 1999–2004 as the reference category and comparing the data of 2005–2010, 2011–2016, 2017–2022 to those of 1999–2004

The results of the quality influence factor analysis are shown in Table 5. Overall, we found that all the RCTs published in CSCD journals that were conducted at multiple centres and that stated the approval of the ethics committee were of better quality. The RCTs that were conducted in teaching hospitals were of better quality with respect to adequate allocation concealment, blinding participants and personnel and other bias. The RCTs with funding were of better quality with respect to random sequence generation, allocation concealment, blinding of participants and personnel, blinding of outcome assessment, incomplete outcome data and other bias. The RCTs reported that the signing of the informed consent form was of better quality for random sequence generation, allocation concealment, incomplete outcome data, selective outcome reporting and other bias.

**Table 5:** Reporting quality for different subgroups of paediatric RCTs in China from 1999–2022

Items	Adequate random sequence generation (*n* = 1272)	Adequate allocation concealment (*n* = 109)	Adequate blinding of participants and personnel (*n* = 150)	Adequate blinding of outcome assessment (*n* = 52)	Incomplete outcome data (low risk) (*n* = 1624)	Selective outcome reporting (low risk) (*n* = 363)	Other bias (low risk) (*n* = 85)
CDCD (*n* = 730)	165 (22.6)	52 (7.1)	52 (7.1)	21 (2.9)	261 (35.8)	114 (15.6)	66 (9.0)
Non-CSCD (*n* = 2815)	1107 (39.3)	57 (2.0)	98 (3.5)	31 (1.1)	1363 (48.4)	249 (8.8)	19 (0.7)
***P *value**	< 0.000	< 0.000	< 0.000	< 0.000	< 0.000	< 0.000	< 0.000
Teaching hospital (*n* = 2781)^a^	988 (35.5)	95 (3.4)	124 (4.5)	44 (1.6)	1283(46.1)	299 (10.8)	80 (2.9)
Non-teaching hospital^a^ (*n* = 654)	253 (38.7)	10 (1.5)	18 (2.8)	5 (0.8) ^b^	290 (44.3)	57 (8.7)	4 (0.6)
***P *value**	0.130	0.012	0.049	0.141	0.408	0.124	< 0.000
Multiple-centre (*n* = 92)	54 (58.7)	29 (31.5)	17 (18.5)	5 (5.4) ^b^	63 (68.5)	23 (25.0)	20 (21.7)
Single-centre (*n* = 3453)	1218 (35.3)	80 (2.3)	133 (3.9)	47 (1.4)	1561 (45.2)	340 (9.8)	65 (1.9)
***P *value**	< 0.000	< 0.000	< 0.000	0.009	< 0.000	< 0.000	< 0.000
Funding (*n* = 539)	216 (40.1)	34 (6.3)	34 (6.3)	15 (2.8)	276 (51.2)	66 (12.2)	33 (6.1)
No funding (*n* = 3006)	1056 (35.1)	75 (2.5)	116 (3.9)	37 (1.2)	1348 (44.8)	297 (9.9)	52 (1.7)
***P *value**	0.028	< 0.000	0.009	0.006	0.006	0.095	< 0.000
Ethical approval (*n* = 661)	384 (58.1)	52 (7.9)	51 (7.7)	19 (2.9)	416 (69.7)	134 (20.3)	77 (11.6)
Not-reported (*n* = 2884)	888 (30.8)	57 (2)	99 (3.4)	33 (1.1)	1163 (40.3)	229 (7.9)	8 (0.3)
***P *value**	< 0.000	< 0.000	< 0.000	0.001	< 0.000	< 0.000	< 0.000
Signed informed Consent (*n* = 1419)	856 (60.3)	77 (5.4)	66 (4.7)	27 (1.9)	848 (59.8)	173 (12.2)	78 (5.5)
Not-reported (*n* = 2126)	416 (19.6)	32 (1.5)	84 (4.0)	25 (1.2)	776 (36.5)	190 (8.9)	7 (0.3)
***P *value**	< 0.000	< 0.000	0.31	0.078	< 0.000	0.002	< 0.000

Data was presented as n (%)

*CSCD* Chinese Science Citation Database,  *RCTs* Randomized controlled trials

^a^We excluded the studies conducted in scientific research institutions

^b^Fisher’s exact test

## Discussion

The number of paediatric RCT trials demonstrated a prominent increase in mainland China, with an average annual growth rate of 7.8%, which was likely related to significant efforts and support from the Chinese government and reflects researchers’ growing interest in paediatric RCTs as the gold standard for evidence-based medicine to guide treatment decisions. We found that the number of RCTs increased obviously in 2011 and began to gradually decrease after 2018, which could be impacted by the COVID-19 pandemic. Previous research reported that the number of RCTs increased annually through 2019 but decreased in 2020 due to the challenges of the coronavirus disease (COVID-19) pandemic. RCTs had to adjust protocols to accommodate the sudden suspension of recruitment, in-person data collection, and safety visits, as well as the delivery of interventions, which may have led to a pause or premature closure of the trial [34].

Overall, 3545 RCTs were from 21 cities, and 32.4% of the RCTs were distributed in the east, while only 247 (7.0%) were distributed in the northwest. Most of the RCTs were initiated by tertiary hospitals, which is consistent with the distribution of economic prosperity, but there is still a large uneven geographical distribution of the sponsors and the research institutions. Additionally, most of the studies analysed paediatric clinical trials conducted in China, and the results were consistent with our previous study [26, 27]. Another previous study also revealed disparities in the global distribution of orthopaedic RCTs, with the majority of the trials originating from the United States, the United Kingdom and Canada, and this global disparity has not improved over the last decade [35]. A lack of funding and language barriers might be possible explanations for this disparity. Therefore, further narrowing regional disparities and improving the clinical trial capability of primary medical institutions are also challenges in China.

Using the Cochrane Collaboration risk of bias tool, we conducted an in-depth analysis of the methodological characteristics and risk of bias of RCTs published in 20 paediatric journals in mainland China from 1999 to 2022. By comparing RCTs published in 2017–2022 with RCTs published in 1999–2004, we found that the quality of RCTs improved in terms of random sequence generation, allocation concealment, blinding of participants and personnel, incomplete outcome data and selective outcome reporting. Overall, there are limited literature reports on the quality assessment of RCTs published in paediatric journals at home and abroad. Compared with a study analysing the quality of paediatric RCTs in China before 2011 [21], the proportions of patients with adequate random sequence generation, allocation concealment and blinding in our study were greater, indicating that the reporting quality of RCTs from paediatric journals has improved, but there is still room for improvement. Compared with those of RCTs in paediatric dentistry and published in foreign journals [36], the proportions of adequate randomization [64.3.0% (117/182) vs. 35.9% (1272/3545)] and blinding [9.3% (17/182) vs. 5.7% (202/3545)] were greater. Compared with those in adult RCTs published in traditional Chinese medicine, the proportions of adequate randomization methods were lower [25.0% (142/579) vs. 35.9% (1272/3545)], whereas the proportions of allocation concealment and blinding were largely greater [26% (151/579) vs. 3.1% (109/3545), 60.0% (349/579) vs. 5.7% (202/3545), respectively] [37].

Given that approximately one-third of the studies involved respiratory disease, we compared the quality of the RCTs on respiratory diseases with those on other diseases (e.g., diseases originating in the perinatal period, digestive diseases), and there were no particular areas or topics of particularly high (or low) quality and only a few studies on COVID-19. Overall, the trends of low risk of bias-related quality assessments of RCTs for respiratory diseases in China improved over time and were consistent with the results in Table 4.

In this study, only 25 RCTs reported all random sequences, allocation concealment methods, blinding methods and complete outcome data, and the study also showed that a large percentage of RCTs showed “unclear” risk of bias. Therefore, there is much room to improve the methodological reporting quality of RCTs in paediatric journals in mainland China, including a detailed description of allocation concealment, which is one of the key factors that makes RCTs the most valuable study design for evaluating the effectiveness of therapeutic interventions [29]. The use of blinding of outcome assessments has often been neglected, as some investigators only mark RCTs as single, double, or triple-blind. We believe that this bias could easily decrease if authors truthfully report why they are not blinded to the outcome assessors to provide readers with a clear understanding of the risk of bias.

Reporting the drop-out rates from RCTs was important to reflect the patient overall assessment of the balance between benefits and harms and to ensure that other recorded outcomes are not biased due to differential drop-out rates and reasons between the treatment arms [38]; however, in this study, only 9.3% of RCTs reported dropouts. We also found that more than half of the trials did not report adverse effects, and the quality of the reported adverse effects did not significantly change over time. In vulnerable children, we should pay more attention to the safety and efficacy of interventions, and any therapeutic effect must be balanced with adverse effects to support a clinical diagnosis by a paediatrician. Moreover, only 2.0% of trials reported sample size calculations performed prior to study initiation, which is substantially low. A lack of sample size calculations before enrolment could lead to an increase in the risk of random errors and reflect statistical significance [39]. Therefore, Chinese paediatric journals, especially non-CSCD journals, should standardize RCT reporting standards, such as reporting the full text in compliance with the reporting criteria CONSORT (Consolidated Standards of Reporting Trials) [40], to reduce the risk of making incorrect conclusions about intervention effects, and Chinese medical journals could learn from the policy of the International Committee of Medical Journal Editors that the information about clinical trial reports should refer to the CONSORT checklists.

Informed consent was an important medical ethical principle to follow in paediatric clinical trials, and a previous study showed that the average consent rate for paediatric randomized controlled trials was 82.6% [41]. We found that RCTs stated that the approval of the ethics committee and the signing of the informed consent form were of better quality, and the reporting quality improved over time; however, approximately 60% of RCTs failed to report whether the study signed an informed consent form. The recommendation should be that journals should require a statement indicating that informed consent was obtained from all participants before study procedures were initiated, or, if consent was not required for a given trial, this should be reported in lieu.

With regard to funding, in this study, most of the included trials were funded by national and provincial resources, and compared with the analysis of RCTs without funding, the reporting quality of RCTs with funding was better, but most trials did not report funding support. A lack of funding is one of the major barriers to conducting paediatric RCTs worldwide [42]. To further facilitate the innovation of paediatric drugs and motivate the efficiency of paediatric clinical trials, the newly revised Drug Administration Law of the People’s Republic of China came into effect on December 1, 2019. It clearly states that it encourages the development and innovation of children’s medicine, supports the development of special medicines that are consistent with children’s physiological characteristics, and prioritizes the approval of children’s medicine [43]. However, previous work suggested that clinical trials with funding may overstate the results or only report favourable findings [42], and how to improve the bias of clinical trials with funding is an important issue worthy of exploration.

There were several limitations in our study: we only studied articles in professional paediatric journals, we may have missed RCTs published in other nonpaediatric-related journals, and we focused only on Chinese RCTs. We may have ignored higher-quality RCTs published by Chinese institutions in international journals or in English, which may cause bias. Given the extremely large number of articles retrieved, we believe that our results are representative. We used the Cochrane Collaboration tool to assess the risk of bias, which can be subjective. We failed to contact the author to resolve any confusion when judging the risk of bias, as we hoped to objectively present the quality of the research report, and two independent authors assessed the included studies to avoid potential biases. At present, many Chinese industry-sponsored paediatric clinical studies have been published in foreign journals, and our study mainly reflects the quality of paediatric RCT trials initiated by Chinese investigators, with only 10 RCT trials subsidized by companies.

## Conclusion

The number and quality of paediatric RCTs reported in China have improved in recent years, but the overall quality was relatively low. Special attention should be given to allocation concealment and blinding outcome assessment, and dropouts, adverse effects and sample size calculations should be reported. Promoting government policies, strengthening the standardization of journal publishing and advancing the registration of clinical trials are feasible measures.

### Supplementary Information

Supplementary Material 1.

Supplementary Material 2.

## Data Availability

The datasets analysed during the current study are available from the following databases in China: China National Knowledge Infrastructure (https://www.cnki.net), China Science and Technology Journal Database (https://qikan.cqvip.com), Wanfang Data Knowledge Service Platform (https://www.wanfangdata.com.cn) and China Biology Medicine disc (www.sinomed.ac.cn). The datasets used and/or analyzed during the current study are available from the corresponding author on reasonable request.
