# Supplementary material for: Quality assessment of paediatric randomized controlled trials published in China from 1999 to 2022: a cross-sectional study
Source: BMC Pediatr. 2024 May 27;24:364. doi: 10.1186/s12887-024-04839-3 (PMC11129423; doi:10.1186/s12887-024-04839-3)
Supplement: Supplementary file 2 — Supplementary Material 2. [file 12887_2024_4839_MOESM2_ESM.docx]

| **Assessment details of the risk of bias ^a^** | | | |
| --- | --- | --- | --- |
| **Domain** | **Low risk of bias** | **High risk of bias** | **Unclear of bias** |
| **Random sequence generation** | The researchers describe a random component in the sequence generation process such as:  • Referring to a random number table;  • Using a computer random number generator;  • Coin tossing;  • Shuffling cards or envelopes;  • Throwing dice;  • Drawing of lots;  • Minimization (minimization may be implemented without a random element, and this is considered to be equivalent to being random). | Sequence generated by odd or even date of birth, rule based on date (or day ) of admission ,based on hospital or clinic record number, etc.  The investigators describe a non-random component in the sequence generation process. Usually, the description would involve some systematic, non-random approach, for example:  • Sequence generated by odd or even date of birth;  • Sequence generated by some rule based on date (or day) of admission;  • Sequence generated by some rule based on hospital or clinic record number.  Some method of non-random categorization of participants, for example:  • Allocation by judgement of the clinician;  • Allocation by preference of the participant;  • Allocation based on the results of a laboratory test or a series of tests;  • Allocation by availability of the intervention. | Insufficient information about the sequence generation process to permit judgement of ‘Low risk’ or ‘High risk’. |
| **allocation concealment** | Participants and investigators enrolling participants could not foresee assignment because one of the following, or an equivalent method, was used to conceal allocation:  • Central allocation (including telephone, web-based and pharmacy-controlled randomization);  • Sequentially numbered drug containers of identical appearance;  • Sequentially numbered, opaque, sealed envelopes. | Participants or investigators enrolling participants could possibly foresee assignments and thus introduce selection bias, such as allocation based on:  • Using an open random allocation schedule (e.g. a list of random numbers);  • Assignment envelopes were used without appropriate safeguards (e.g. if envelopes were unsealed or non-opaque or not sequentially numbered);  • Alternation or rotation.  • Date of birth;  • Case record number;  • Any other explicitly unconcealed procedure. | Insufficient information to permit judgement of ‘Low risk’ or ‘High risk’. Concealment is not described or not described in sufficient detail to allow a definite judgement. |
| **Blinding of participants and personnel** | Any one of the following:  • No blinding or incomplete blinding, but the review authors judge that the outcome is not likely to be influenced by lack of blinding;  • Blinding of participants and key study personnel ensured, and unlikely that the blinding could have been broken. | Any one of the following:  • No blinding or incomplete blinding, and the outcome is likely to be influenced by lack of blinding;  • Blinding of key study participants and personnel attempted, but likely that the blinding could have been broken, and the outcome is likely to be influenced by lack of blinding. | Any one of the following:  • Insufficient information to permit judgement of ‘Low risk’ or ‘High risk’;  • The study did not address this outcome. |
| **Blinding of outcome assessment** | Any one of the following:  • No blinding of outcome assessment, but the review authors judge that the outcome measurement is not likely to be influenced by lack of blinding;  • Blinding of outcome assessment ensured, and unlikely that the blinding could have been broken. | Any one of the following:  • No blinding of outcome assessment, and the outcome measurement is likely to be influenced by lack of blinding;  • Blinding of outcome assessment, but likely that the blinding could have been broken, and the outcome measurement is likely to be influenced by lack of blinding. | Any one of the following:  • Insufficient information to permit judgement of ‘Low risk’ or ‘High risk’;  • The study did not address this outcome. |
| **Incomplete outcome data** | Any one of the following:  • No missing outcome data;  • Reasons for missing outcome data unlikely to be related to true outcome (for survival data, censoring  unlikely to be introducing bias);  • Missing outcome data balanced in numbers across intervention groups, with similar reasons for missing data across groups;  • For dichotomous outcome data, the proportion of missing outcomes compared with observed event risk not enough to have a clinically relevant impact on the intervention effect estimate;  • For continuous outcome data, plausible effect size (difference in means or standardized difference in means) among missing outcomes not enough to have a clinically relevant impact on observed effect size;  • Missing data have been imputed using appropriate methods. | Any one of the following:  • Reason for missing outcome data likely to be related to true outcome, with either imbalance in numbers or reasons for missing data across intervention groups;  • For dichotomous outcome data, the proportion of missing outcomes compared with observed event risk enough to induce clinically relevant bias in intervention effect estimate;  • For continuous outcome data, plausible effect size (difference in means or standardized difference in means) among missing outcomes enough to induce clinically relevant bias in observed effect size;  • ‘As-treated’ analysis done with substantial departure of the intervention received from that assigned at randomization;  • Potentially inappropriate application of simple imputation. | Any one of the following:  • Insufficient reporting of attrition/exclusions to permit judgement of ‘Low risk’ or ‘High risk’ (e.g. number randomized not stated, no reasons for missing data provided);  • The study did not address this outcome. |
| **Selective reporting** | Any of the following:  • The study protocol is available and all of the study’s pre-specified (primary and secondary) outcomes that  are of interest in the review have been reported in the pre-specified way;  • The study protocol is not available but it is clear that the published reports include all expected outcomes, including those that were pre-specified (convincing text of this nature may be uncommon). | Any one of the following:  • Not all of the study’s pre-specified primary outcomes have been reported;  • One or more primary outcomes is reported using measurements, analysis methods or subsets of the data (e.g.  subscales) that were not pre-specified;  • One or more reported primary outcomes were not pre-specified (unless clear justification for their reporting  is provided, such as an unexpected adverse effect);  • One or more outcomes of interest in the review are reported incompletely so that they cannot be entered in a  meta-analysis;  • The study report fails to include results for a key outcome that would be expected to have been reported for such a study. | Insufficient information to permit judgement of ‘Low risk’ or ‘High risk’. It is likely that the majority of studies will fall into this category. |
| **Other bias** | The study appears to be free of other sources of bias. | There is at least one important risk of bias. For example, the study:  • Had a potential source of bias related to the specific study design used; or  • Has been claimed to have been fraudulent; or  • Had some other problem. | There may be a risk of bias, but there is either:  • Insufficient information to assess whether an important risk of bias exists; or  • Insufficient rationale or evidence that an identified problem will introduce bias. |
| ^a^: Evaluation of the quality of the research methods was based on the Cochrane Handbook for Systematic Reviews of Interventions (Version 5.1.0)  ^[34]^ | | | |
